# Identification of Hub Genes and Key Pathways Associated with Follicular Lymphoma

**DOI:** 10.1155/2022/5369104

**Published:** 2022-07-31

**Authors:** Qing Zhang, Meng Wang

**Affiliations:** Yangtze University Health Science Center, Jingzhou 434020, China

## Abstract

Follicular lymphoma (FL) is the second most prevalent form of non-Hodgkin lymphoma (NHL) and accounts for almost 20% of all NHL cases. Although FL patients' overall survival rates have steadily increased, there is still no accepted standard of care for individuals who experience recurrence or resistance to treatment. Hence, it is needed to evaluate the precise molecular cascades underlying FL to develop efficient diagnostic and treatment approaches. Herein, we aimed to evaluate variations in gene expression profiles, explore the underlying mechanisms, and find new FL targets. In the present study, Gene Expression Omnibus (GEO) database was employed to evaluate microarray datasets including GSE32018 and GSE55267. *R* software was employed to evaluate differentially expressed genes (DEGs) between FL and noncancer samples. The DEGs were evaluated using GO, KEGG pathway enrichment analysis, and PPI network to evaluate hub genes, which were then, examined using gene function enrichment analysis. According to the obtained results, a total of 190 upregulated and 162 downregulated DEGs were evaluated. Following the generation of PPI networks, 15 hub genes in highly connected upregulated DEGs were selected including FN1, MMP9, CCL2, CD8A, POSTN, CCR5, COL3A1, CXCL12, VCAM1, COL1A2, CCL5, SPARC, TIMP1, CXCL9, and IL18. The GO enrichment evaluation of the underlined hub genes indicated that the immunological response was the most considerably enriched term. Twelve significant cascades were found using the KEGG pathway analysis, most of which were linked to cellular structure and immunity. Our findings suggested that FN1, SPARC, POSTN, MMP9, and VCAM1 genes are potential biomarkers of FL, and cellular immunity contributes to the pathogenesis of FL. Moreover, the unique DEGs and cascades found in the present study may present new perspectives on the molecular basis of FL's underlying mechanisms as well as a new understanding of FL's future precise management.

## 1. Introduction

Follicular lymphoma (FL) is the second most prevalent subtype of NHL, accounting for approximately 20% of all NHL cases [1, 2]. The clinical signs of FL can vary since it is an indolent lymphoma. The majority of FL cases are asymptomatic but some develop noticeable symptoms, for example, multisite lymphoid tissue invasion [3]. Many patients are diagnosed at advanced stages due to the absence of particular symptoms and techniques for earlier detection, which is therefore linked to poor outcomes. Generally, indolent lymphomas are less severe if left untreated, but they are also more difficult to treat due to their reduced proliferative potential, which renders them resistant to treatment. The anti-CD20 monoclonal antibody rituximab, along with other advances in treatment, has altered the survival outcomes of FL during the past few decades, leading to more hopeful results for patients. Despite the abovementioned advances in survival, FL remains a highly heterogeneous disease with varying results. In most patients, the disease develops lazily and can be relieved for a long time after treatment, while in others, the disease shows more aggressive and chemotherapy-resistant behavior [4, 5]. Therefore, it is needed to explore the specific molecular cascades responsible for FL to develop promising diagnostic and therapeutic approaches.

The emergence of bioinformatics technology offers new approaches to investigating the molecular basis of disease and identifying biomarkers, which encourages the advancement of tumor molecular diagnostics, targeted and individualized treatment, and prediction of prognosis [6]. In this study, hub genes linked to the pathogenesis of FL were investigated using bioinformatics tools. Hub genes and putative mechanisms, such as signaling cascades linked to FL may help us better understand its pathogenesis and, as a result, give us new information for FL's future management.

## 2. Materials and Methods

### 2.1. Microarray Data

The Gene Expression Omnibus (GEO) database (http://www.ncbi.nlm.nih.gov/geo/) was used to retrieve the datasets by specifying “follicular lymphoma” as the keyword, “Homo sapiens” like the organism, and “expression profiling by array” as the study type. The final datasets chosen were GSE32018 [7] and GSE55267 [8] which contained data from both FL and noncancerous samples. GSE32018 included 23 FL and 13 noncancerous samples, and the gene detection platform was GPL6480 Agilent-014850 Whole Human Genome Microarray 4 × 44 K G4112 F (Agilent Technologies, Palo Alto, CA, USA). The GSE55267 expression profile comprised 63 FL and 6 noncancerous samples; the gene platform was GPL570 [HG-U133 Plus 2] Affymetrix Human Genome U133 Plus 2.0 Array (Affymetrix, Santa Clara, CA, USA). This study did not involve ethical guidelines as all the data were freely accessible.

### 2.2. Evaluation Criteria of DEGs

Based on the acquired platform annotation files, Perl transformed the original probe-level data in the Series Matrix Files into gene symbols. The “SVA” program in R software (version 4.1.0) was utilized for batch correction of the datasets because variations in instrument types, technical proficiency of experimenters, and reagents may result in batch changes in experimental outcomes. The average expression value was used to choose the expression values of several probes for a single gene. The variations in gene expression were evaluated between FL and noncancerous samples using the “Limma” package in R. *p* < 0.05 and a log2 fold change (log2FC) absolute value greater than 1 were the screening conditions for DEGs. Next, a volcano graph was developed to depict the up- and downregulated genes. The considerably upregulated genes were evaluated for further evaluation.

### 2.3. Functional Annotation of DEGs

KEGG (Kyoto Encyclopedia of Genes and Genomes) pathway analysis was employed to predict the cellular cascades responsible for the variations in DEGs. The R tool “Clusterprofiler” was employed to evaluate the data obtained from Gene Ontology (GO) and KEGG pathways (the screening criteria: *p* < 0.05, and *p* < 0.05 FDR). The R package “Pathview” was employed for visualizing the filtered key terms, followed by representing them in a histogram.

### 2.4. Development of PPI Network and Evaluation of Hub Genes

By using the Search Tool for the Retrieval of Interacting Genes/Proteins (STRING, https://string-db.org), a protein-protein interaction (PPI) network was generated to find the hub regulating genes and explore the link between the DEGs. These genes needed nodes with a degree  ≥1 and an interaction score of  ≥0.04. The top 15 PPI network genes, which were determined to be hub genes, were evaluated using density analysis through R's “Barplot” function. The hub genes were subjected to a preliminary examination with systematic and extensive biological function notes using the Database for Annotation, Visualization, and Integrated Discovery (DAVID, https://David.Ncifcrf.gov/).

## 3. Results

### 3.1. Evaluation of DEGs

**Figure 1:**
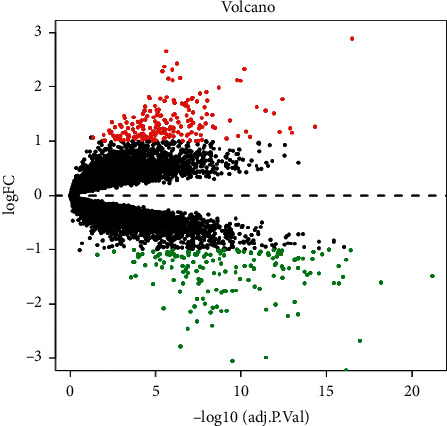
The volcano diagram reveals the DEGs between FL and noncancerous samples. Here, the red and green spots designate upregulated and downregulated genes, respectively. DEGs, differentially expressed genes; FL, follicular lymphoma.

A total of 352 DEGs, comprising 190 upregulated and 162 downregulated genes, were retrieved from the GSE55267 and GSE32018, as depicted in Figure 1.

### 3.2. DEG Enrichment Analysis Using GO and KEGG

**Figure 2:**
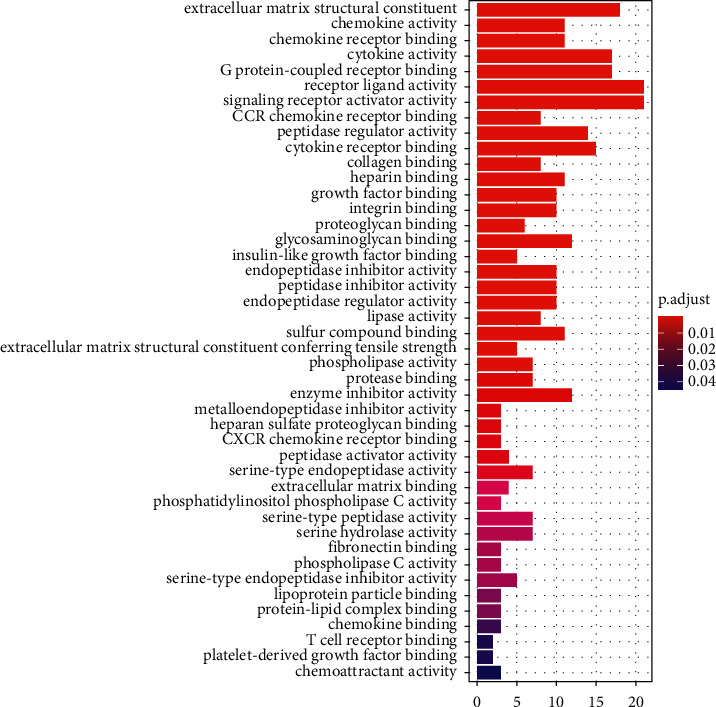
The results obtained from GO enrichment analysis for upregulated DEGs. Histogram color from red to purple indicates a gradual increase in *p* value, red indicates *p* < 0.01, and purple indicates *p* > 0.04. DEGs, differentially expressed genes; GO, Gene Ontology.

**Figure 3:**
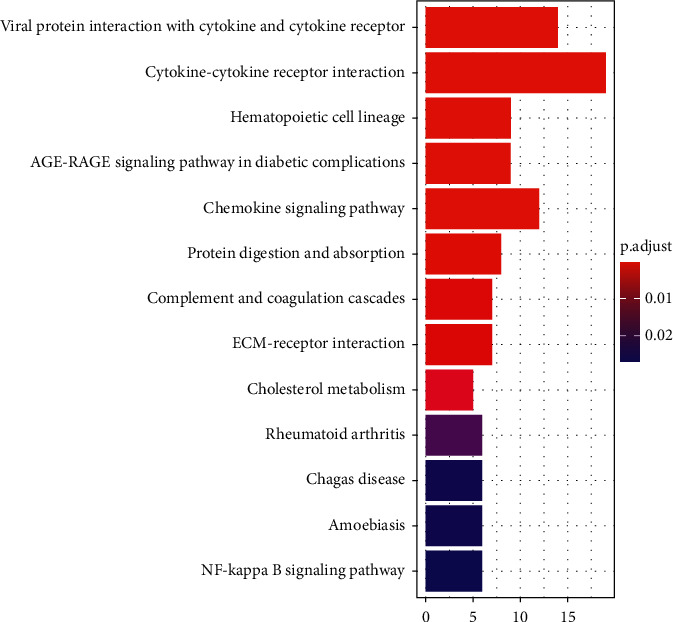
KEGG enrichment analysis of upregulated DEGs. Histogram color from red to purple indicates a gradual increase in *p* value, red indicates *p* < 0.01, and purple indicates *p* > 0.02. DEGs, differentially expressed genes; ECM, extracellular matrix; KEGG, Kyoto Encyclopedia of Genes and Genomes.

The DAVID version 123 database (https://David.Ncifcrf.gov/) was employed for the GO and KEGG enrichment analyses in order to obtain the biological data from 190 upregulated DEGs. The 44 items in the GO enrichment analysis (Figure 2) showed receptor ligand activity, signaling receptor activity, the structural composition of the extracellular matrix (ECM), cytokine activity, G protein-coupled receptor (GPCR) binding, and other molecular functions were primarily enriched. The data obtained from the KEGG enrichment analysis indicated that DEGs were considerably enriched in the interactions between the ECM and receptors (Figure 3), the chemokine signal cascade, and viral proteins that interact with cytokines and cytokine receptors.

### 3.3. PPI Network Construction and Hub Gene Identification

**Figure 4:**
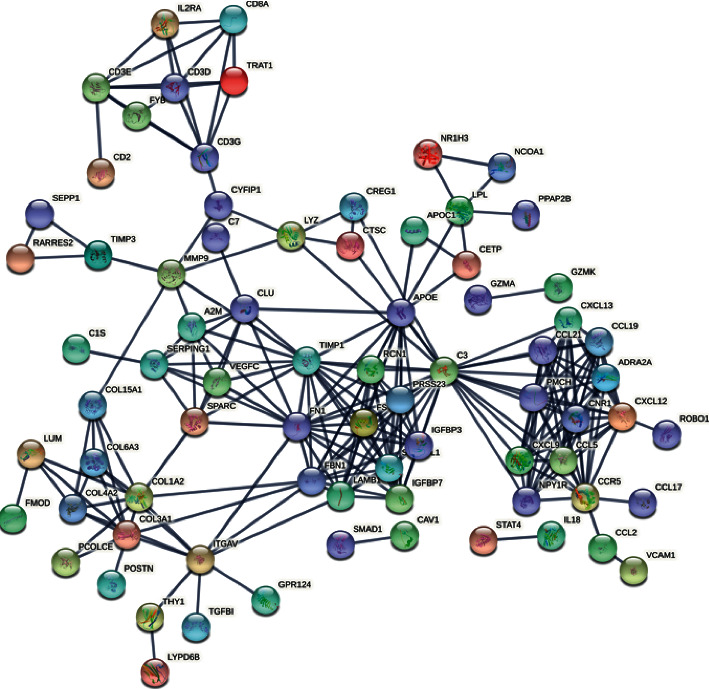
The generation of the PPI network having the upregulated DEGs. DEGs and PPI indicate differentially expressed genes, and protein-protein interaction, accordingly.

**Figure 5:**
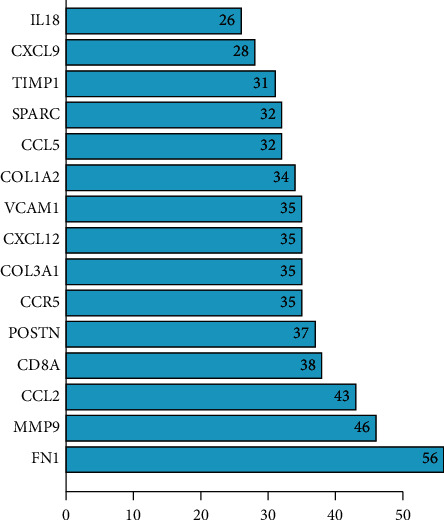
Top 15 hub genes with a high degree of connectivity.

**Figure 6:**
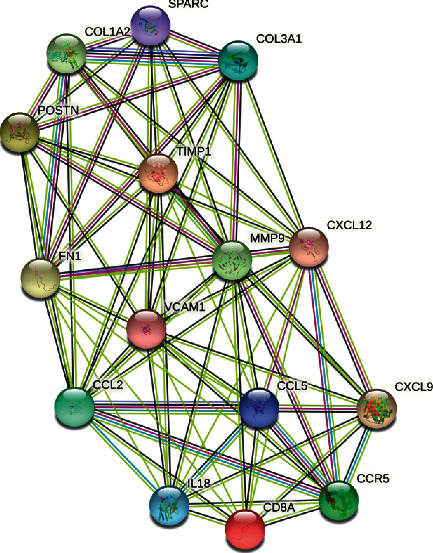
PPI network of the hub genes.

To evaluate the roles of DEGs, the STRING for proteins encoded by DEGs was searched, followed by the construction of a PPI network with 829 edges and 159 nodes (Figure 4). In this network, 15 hub genes were obtained: fibronectin 1 (FN1), C–C motif chemokine ligand 5 (CCL5), C–C motif chemokine ligand 2 (CCL2), matrix metallopeptidase 9 (MMP9), CD8A molecule (CD8A), periostin (POSTN), C-X-C motif chemokine ligand 9 (CXCL9), C–C motif chemokine receptor 5 (CCR5), vascular cell adhesion molecule 1 (VCAM1), collagen type III alpha 1 chain (COL3A1), collagen type I alpha 2 chains (COL1A2), secreted protein acidic and cysteine-rich (SPARC), C-X-C motif chemokine ligand 12 (CXCL12), TIMP metallopeptidase inhibitor 1 (TIMP1), and interleukin 18 (IL18) (Figure 5 and 6).

### 3.4. GO and KEGG Enrichment Analysis of the Hub Genes

The results of the GO analysis were provided from three perspectives, as shown in Table 1: biological process (BP), cellular component (CC), and molecular function (MF). The ECM organization, inflammatory response, and immunological response were the most enriched GO terms associated with BP. Extracellular space, extracellular area, and extracellular exosomes were primarily linked to CC. The major function of MF was to bind proteins. The hub genes were enriched in several pathways, including cytokine-cytokine receptor interaction, chemokine signaling cascades, ECM-receptor interaction, and others, according to the KEGG data.  Table 2 lists the hub gene obtained from the KEGG pathway enrichment study.

## 4. Discussion

FL is a malignant lymphohematopoietic tumor that develops from germinal center B cells [9]. It is still regarded as an incurable malignant tumor despite having a median overall survival duration of 15–20 years [10]. Therefore, identifying the FL hub gene is crucial for understanding the possible molecular mechanisms underlying FL susceptibility and progression. An efficient method for identifying prospective diagnostic biomarkers and therapeutic targets in the prevention and treatment of FL is to use bioinformatics evaluation, which is a powerful tool for understanding the molecular cascades behind disease onset and progression.

Herein, we selected two accessible microarray datasets, GSE32018 and GSE55267, and used R to evaluate 190 upregulated DEGs according to the inclusion criteria between 86 FL and 19 noncancerous samples. The GO and KEGG pathway enrichment analyses as well as the PPI network were extensively processed to find hub genes among the upregulated DEGs. The hub genes were most abundant in KEGG signaling cascades including the NOD-like receptor signaling cascades, chemokine signaling cascade, cytokine-cytokine receptor signaling cascade, and TNF signaling cascade. One of the features of lymphomagenesis is immunological dysregulation, and cytokines are the fundamental secretory proteins of inflammation, cellular communication, and immune control. Herein, disruption of the cytokine balance may be a crucial event in the susceptibility to FL [11]. The underlined data showed consistency with the results obtained from other studies [12–15]. Other signaling pathways such as the interaction of ECM-receptor, leukocyte transendothelial migration, and focal adhesion are also associated. However, the mechanism by which these pathways affect the pathogenesis of FL is not clearly understood. According to the reported study by Li et al. [16], the mechanism of multiple DEGs candidate biomarkers predicting the metastatic epithelial ovarian carcinoma (EOC) prognosis may be related to the ECM-receptor interaction. Esophageal squamous cell carcinoma (ESCC) is considered to be one of the most prevalent malignancies of the digestive tract. According to the reported studies, the ECM-receptor interaction cascade is involved in ESCC metastasis [17]. During inflammation, leukocytes are activated by chemokines, transported to the site of injury, adhered to vascular endothelial cells, and then moved along the wall to the endothelial boundary, followed by migrating through the endothelium basement membrane. The overall process is called transendothelial migration [18]. Multiple invasive solid tumors and metastases are distinguished by their overexpression of focal adhesion kinase (FAK). A study revealed the predictive potential of FAK in diffuse large B-cell lymphoma. The multivariate Cox analysis revealed that lower FAK expression may be an independent predictor of poor disease outcomes [19]. Furthermore, another study reported that leukocyte transendothelial migration, ECM-receptor interaction, and focal adhesion were implicated in the development of diffuse large B-cell lymphoma [20].

The PPI network was constructed to gain a comprehensive insight into the functional relationships between DEGs and identify the 15 genes (*i.e.,* FN1, MMP9, CCL2, CD8A, POSTN, CCR5, COL3A1, CXCL12, VCAM1, COL1A2, CCL5, SPARC, TIMP1, CXCL9, and IL18) that served as the network's hubs. In addition to cytokines involved in multiple signal pathways, FN1, SPARC, POSTN, MMP9, and VCAM1 are mainly enriched in ECM-receptor interaction, ECM organization, and cell adhesion.

The extracellular matrix glycoprotein FN1 mediates a wide range of cellular interactions with the ECM. It is associated with multiple cellular processes such as cell adhesion, migration, wound healing, and blood coagulation [21]. SPARC belongs to the family of matricellular proteins and is involved in extracellular matrix deposition and tissue remodeling [22]. In cancer models, SPARC has been shown to affect ECM components, cell adhesion, tumor growth, migration, apoptosis, and chemosensitivity. It can also promote cell invasion by inducing epithelial-mesenchymal transition (EMT) to increase cell motility in different cancers [23–25]. However, SPARC expression provides different outcomes depending on the cancer type and its stages [26]. Li et al. [27] sequenced 15 pairs of gastric adenocarcinoma tumor tissues and adjacent tissues. Their obtained result demonstrated that the FN1 expression and SPARC in gastric adenocarcinoma tissues were found to be closely correlated to their poor prognosis. Bao et al. and Song et al. [22, 28] also revealed that the expression levels of FN1 and SPARC were considerably linked with diffuse large B-cell lymphoma. POSTN is a stromal cell protein with a molecular weight of 93 kDa, which have vital functions in bone development, maturation, repair, and EMT [29]. By binding with its cell surface receptor integrins, POSTN can control the growth, angiogenesis, invasion, and metastasis of cancerous cells. Moreover, this also affects intracellular signal transduction [30].

MMP9 is an intracellular zinc-dependent and membrane-bound endopeptidase. It can contribute to the degradation of ECM proteins (including collagen, elastin, and laminin) and the remodeling of ECM in various physiological and pathological processes [31]. In 1989, it was found that VCAM1, a key member of the immunoglobulin superfamily, is an endothelial cell adhesion receptor that contributes to the onset and spread of inflammatory disorders, especially the transendothelial migration process [32, 33]. Several studies have confirmed that POSTN, MMP9, and VCAM1 are implicated in regulating the incidence and development of solid tumors such as lung cancer [34, 35], gastric cancer [36, 37], and rectal cancer [32, 38].

In conclusion, the findings reported here suggest that FN1, SPARC, POSTN, MMP9, and VCAM1 considerably contribute to the pathophysiology of FL. However, additional in vitro and in vivo research is required to confirm the role of these gene-regulated molecular networks in FL. Moreover, further research is needed to explore the patterns of gene expression in various stages of FL which may result in the evaluation of candidate biomarkers for accurate diagnosis and effective therapeutic strategies.

## Figures and Tables

**Table 1:** Considerably enriched GO terms of Hub Genes.

GO ID	Term	*P* value	FDR	Genes
Biological process				
GO:0006955	Immune response	6.07E-07	4.25E-05	CXCL9, CXCL12, CD8A, CCL5, IL18, CCL2, CCR5
GO:0030198	Extracellular matrix organization	3.78E-07	3.53E-05	COL3A1, POSTN, VCAM1, SPARC, COL1A2, FN1
GO:0006954	Inflammatory response	9.66E-06	4.51E-04	CXCL9, CXCL12, CCL5, IL18, CCL2, CCR5
GO:0060326	Cell chemotaxis	1.99E-07	3.53E-05	CXCL9, CXCL12, VCAM1, CCL5, CCL2
GO:0070098	Chemokine-mediated signaling pathway	2.84E-07	3.53E-05	CXCL9, CXCL12, CCL5, CCL2, CCR5
GO:0006935	Chemotaxis	2.51E-06	1.41E-04	CXCL9, CXCL12, CCL5, CCL2, CCR5
GO:0007155	Cell adhesion	4.44E-04	0.00730519	POSTN, CXCL12, VCAM1, FN1, CCL2
GO:0007186	G protein-coupled receptor signaling pathway	0.005304452	0.047911184	CXCL9, CXCL12, CCL5, CCL2, CCR5
Cellular component				
GO:0005615	Extracellular space	2.01E-12	9.24E-11	POSTN, CXCL9, VCAM1, SPARC, IL18, FN1, MMP9, COL3A1, CXCL12, COL1A2, CCL5, CCL2, TIMP1
GO:0005576	Extracellular region	7.02E-10	1.61E-08	COL3A1, CXCL9, CXCL12, SPARC, COL1A2, CD8A, CCL5, IL18, FN1, CCL2, TIMP1, MMP9
GO:0070062	Extracellular exosome	0.013159982	0.075669898	CXCL12, VCAM1, COL1A2, IL18, FN1, TIMP1, MMP9
GO:0005578	Proteinaceous extracellular matrix	1.19E-06	1.82E-05	POSTN, SPARC, COL1A2, FN1, TIMP1, MMP9
GO:0009897	External side of plasma membrane	1.66E-05	1.91E-04	CXCL9, CXCL12, VCAM1, CD8A, CCR5
Molecular function				
GO:0005515	Protein binding	0.040126286	0.137098144	COL3A1, POSTN, CXCL9, SPARC, COL1A2, CD8A, CCL5, IL18, FN1, TIMP1, CCR5, MMP9

FDR, false discovery rate; GO, gene ontology; ID, identity document.

**Table 2:** Considerably enriched KEGG terms of hub genes.

KEGG ID	Term	*P* value	FDR	Gene
hsa04060	Cytokine-cytokine receptor interaction	3.41E-05	0.001398803	CXCL9, CXCL12, CCL5, IL18, CCL2, CCR5
hsa04062	Chemokine signaling pathway	2.16E-04	0.004430241	CXCL9, CXCL12, CCL5, CCL2, CCR5
hsa05323	Rheumatoid arthritis	4.09E-04	0.005595854	CXCL12, CCL5, IL18, CCL2
hsa04668	TNF signaling pathway	7.27E-04	0.007450133	VCAM1, CCL5, CCL2, MMP9
hsa05144	Malaria	0.003134748	0.025704934	VCAM1, IL18, CCL2
hsa04621	NOD-like receptor signaling pathway	0.004077271	0.027861351	CCL5, IL18, CCL2
hsa04512	ECM-receptor interaction	0.009611516	0.05629602	COL3A1, COL1A2, FN1
hsa05146	Amoebiasis	0.014037109	0.071940185	COL3A1, COL1A2, FN1
hsa04670	Leukocyte transendothelial migration	0.01639082	0.074669289	CXCL12, VCAM1, MMP9
hsa05164	Influenza A	0.035546833	0.145742015	CCL5, IL18, CCL2
hsa04510	Focal adhesion	0.048349706	0.180212542	COL3A1, COL1A2, FN1
hsa05143	African trypanosomiasis	0.056115365	0.191727498	VCAM1, IL18

ECM, extracellular matrix; FDR, false discovery rate; ID, identity document; NOD, nucleotide-binding oligomerization domain; KEGG, Kyoto Encyclopedia of Genes and Genomes; TNF, tumor necrosis factor.

## Data Availability

The data used to support the findings of this study are available from the corresponding author upon request.

## List of abbreviations

BP: Biological process
CC: Cellular component
DEGs: Differentially expressed genes
ECM: Extracellular matrix
EMT: Epithelial-mesenchymal transition
FAK: Focal adhesion kinase
FDR: False discovery rate
FL: Follicular lymphoma
FN1: Fibronectin 1
GEO: Gene expression omnibus
GO: Gene Ontology
KEGG: Kyoto Encyclopedia of Genes and Genomes
MF: Molecular function
MMP9: Matrix metallopeptidase 9
POSTN: Periostin
PPI: Protein-protein interaction
SPARC: Secreted protein acidic and cysteine-rich
TIMP1: TIMP metallopeptidase inhibitor 1
VCAM1: Vascular cell adhesion molecule 1.
